# Effects of low-dose esketamine on hypoxemia during gastroscopy in patients with moderate-to-high risk obstructive sleep apnea: protocol for a prospective, randomized, controlled trial

**DOI:** 10.1186/s13063-025-09098-w

**Published:** 2025-09-26

**Authors:** Xin-Ming Li, Si-Qi Hao, Xiu-Ru Qi, Dan-Dan Hao, Ying Li, Li-Xin An

**Affiliations:** Department of Anesthesiology, Beijing Friendship Hospital, Capital Medical University, No. 95 Yongan Road, Xicheng District, Beijing, 100050 China

**Keywords:** Esketamine, Obstructive sleep apnea, Procedural sedation, Hypoxemia, Painless gastroscopy

## Abstract

**Background:**

Propofol sedation in patients with Obstructive Sleep Apnea (OSA) frequently induces hypoxemia, posing significant clinical risks. Esketamine, an N-Methyl-D-Aspartate (NMDA) receptor antagonist, may reduce propofol requirements while preserving respiratory stability, but its efficacy in OSA patients remains unproven. At the studied dose (0.25 mg/kg), esketamine’s potential side effects (transient hypertension) are expected to be mild and self-limited. Therefore, we aimed to test whether low-dose esketamine (0.25 mg/kg) can reduce the incidence of hypoxemia in moderate-to-high risk OSA patients during propofol-based painless gastroscopy.

**Methods:**

This single-center, double-blind, randomized controlled superiority trial will enroll 294 patients (STOP-Bang score ≥ 3, 18–90 years, STOP-Bang = Snoring, Tiredness, Observed apnea, Pressure [blood], Body Mass Index [BMI], Age, Neck size, Gender.) undergoing gastroscopy. Participants will be randomized 1:1 to receive either esketamine (0.25 mg/kg) plus propofol or saline placebo plus propofol, stratified by age (18–65 vs. > 65 years) and OSA severity (STOP-Bang 5–6 vs. ≥ 7). The primary outcome is the incidence of hypoxemia (Peripheral Oxygen Saturation [SpO_2_] < 90% for > 10 s). Secondary outcomes include severe hypoxemia (SpO_2_ ≤ 75% or ≤ 90% for ≥ 60 s), duration of hypoxemia, emergency airway management, propofol consumption, hemodynamic stability, involuntary body movements, procedure/recovery times, and clinician satisfaction (measured via 10-cm Visual Analog Scale [VAS]).

**Discussion:**

This protocol rigorously evaluates esketamine’s potential to improve sedation safety in OSA patients, addressing a critical gap in peri-procedural care.

**Trial registration:**

Chinese Clinical Trial Registry (ChiCTR2500099420). Registered on March 24, 2025 (Supplementary File 2). Si-Qi Hao is a co-first author with the same contribution as the first author. The corresponding author is Li-Xin An.

## Administrative information

**Table:** 

Title {1}	Effects of Low-Dose Esketamine on Hypoxemia During Gastroscopy in Patients with Moderate-to-High Risk Obstructive Sleep Apnea: Protocol for a Prospective, Randomized, Controlled Trial
Trial registration {2a and 2b}.	Chinese Clinical Trial Registry (ChiCTR2500099420), registered on 24 March, 2025. httSi-Qi Hao is a co-first author with the same contribution as the first author. The corresponding author is Li-Xin Anp://www.chictr.org.cn
Protocol version {3}	Version 3.0, dated July 28, 2025
Funding {4}	This study is supported by the Beijing Tongzhou District Science and Technology Project (Grant No. WS2024036). The funder has no role in study design, execution, or publication.
Author details {5a}	Affiliations and contact information provided above. The trial will be conducted by a multidisciplinary team. The Chief Investigator (LXA) oversees all scientific aspects. Co-first authors (QXR, LXM) manage daily operations and data collection. An independent statistician performs randomization and analysis. The Monthly Data and Safety Monitoring Board (DSMB) monitors safety. Endoscopists (≥500 procedures) perform gastroscopy under standardized protocols. The funder has no role in trial conduct.
Name and contact information for the trial sponsor {5b}	Name: Science and Technology Commission of Tongzhou District, Beijing Municipality. Contact: Tel: 0086-10-89526651, Email: kwzhkl@bjtzh.gov.cn
Role of sponsor {5c}	The sponsor has no role in study design, data collection, analysis, or publication.

## Introduction

### Background and rationale {6a}

Gastric cancer is among the most prevalent and lethal malignancies globally [1]. Early detection through screening can improve five-year survival rates to approximately 70%, while advanced-stage disease carries a significantly poorer prognosis of about 20% survival [2, 3]. Gastroscopy serves as the gold standard for both screening and early diagnosis of gastric neoplasms. Propofol sedation, while improving patient comfort, frequently induces hypoxemia and hypotension [4], complications exacerbated in patients with moderate-to-high risk OSA (STOP-Bang score ≥ 3) [5, 6].

The pathophysiology of sedation-associated hypoxemia in these patients involves multiple factors: anatomical airway narrowing, increased thoracic negative pressure, gastroesophageal reflux, chronic pharyngitis, and excessive sedative dosing. OSA has been independently associated with hypoxemia during endoscopic procedures [7], and its prevalence in the general population ranges from 2 to 26% [8]. These findings underscore the critical importance of optimal airway management and judicious sedative selection in this vulnerable population.

The ideal sedative adjunct should simultaneously reduce propofol requirements while minimizing adverse effects on oxygenation and hemodynamics, while still maintaining excellent procedural conditions. Current alternatives present significant limitations: midazolam and opioids exacerbate respiratory and cardiovascular depression [9]; ketamine may induce psychotomimetic effects [10]; dexmedetomidine frequently causes prolonged hypotension and bradycardia [11, 12]; and intravenous lidocaine carries toxicity risks [13].

Esketamine, the S-enantiomer of ketamine, offers several pharmacological advantages as an NMDA receptor antagonist. It provides synergistic sedation and analgesia while exhibiting minimal respiratory depression [14, 15]. Clinical studies demonstrate its propofol-sparing effects and excellent safety profile during urological procedures in elderly patients [16]. At the studied dose (0.25 mg/kg), its potential side effects—including transient hypertension, tachycardia, and emergence phenomena—are typically mild and self-limited [17]. However, evidence regarding esketamine’s efficacy in preventing hypoxemia during gastroscopy in moderate-to-high risk OSA patients remains limited.

#### Objectives {7}

This trial aims to determine whether intravenous low-dose esketamine (0.25 mg/kg) reduces the incidence of hypoxemia (defined as SpO_2_ < 90% for > 10 s) by ≥ 15% compared to placebo during propofol-sedated gastroscopy in patients with moderate-to-high risk OSA (STOP-Bang score ≥ 3), using a one-sided *α* = 0.025.

##### Trial design {8}

**Fig. 1:**
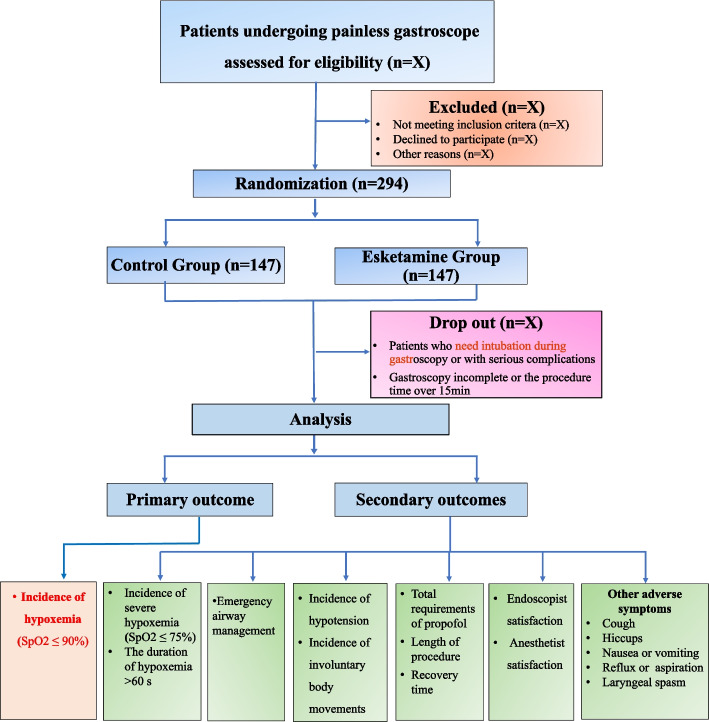
CONSORT diagram

This single-center, prospective, double-blind, randomized, superiority trial will evaluate whether low-dose esketamine (0.25 mg/kg) reduces hypoxemia incidence compared to placebo during propofol-sedated gastroscopy in patients with moderate-to-high OSA risk (STOP-Bang score ≥ 3).We will enroll 294 participants, randomly allocating them in a 1:1 ratio to either Esketamine group (0.25 mg/kg esketamine + propofol) or Control group (equal-volume saline + propofol). The study duration is 21 months. Eligible patients will be screened according to inclusion/exclusion criteria, with baseline characteristics recorded in case report forms (CRFs). Continuous monitoring of vital signs will occur from gastroscopy room admission through Post-Anesthesia Care Unit (PACU) recovery, with all interventions and assessments standardized (Fig. 1).

## Methods: participants, interventions, and outcomes

### Study setting {9}

This single-center trial will be conducted at the Digestive Endoscopy Center of Beijing Friendship Hospital, Affiliated Capital Medical University.

### Eligibility criteria {10}

Inclusion criteria are as follows: (1) adults aged 18–80 years scheduled for elective painless gastroscopy; (2) American Society of Anesthesiologists (ASA) Physical Status I–III; (3) moderate-to-high risk of OSA (STOP-Bang score ≥ 3).

Exclusion criteria are as follows: (1) inability to provide informed consent or cooperate with study procedures; (2) allergic to esketamine or other sedatives; (3) active respiratory infection (pharyngitis, tonsillitis, or upper respiratory tract infection) within 7 days; (4) current pneumonia or room air SpO_2_ < 90%; (5) severe neurological or psychiatric disorders; (6) recent use (within 7 days) of sedatives, analgesics, or hypnotics; (7) uncontrolled hypertension—systolic blood pressure (SBP) > 160 mmHg or diastolic blood pressure (DBP) > 100 mmHg despite treatment; (8) history of malignant arrhythmias; (9) untreated or poorly controlled hyperthyroidism; (10) pregnancy or lactation; (11) participation in another clinical trial within 3 months.

Withdrawal criteria are as follows: (1) requirement for tracheal intubation; (2) serious adverse events or clinical complications; (3) incomplete gastroscopy or procedure duration > 15 min; (4) investigator determination that continuation would compromise patient safety.

Participant Rights: All participants retain the right to withdraw from the study at any time without penalty. Reasons for withdrawal will be documented in CRFs.

Intervention Provider Qualifications: Board-certified anesthesiologists must have ≥ 5 years of procedural sedation experience. Board-certified endoscopists must have performed ≥ 500 gastroscopy procedures, less than 1000.

Statistical considerations are as follows: (1) Primary analysis will follow intention-to-treat (ITT) principles. (2) Secondary per-protocol analysis will include only compliant cases. (3) A fixed anesthesia team will maintain procedural consistency.

The study workflow will include the following: (1) Participant screening—Potential participants will undergo preliminary eligibility assessment at Anesthesiology Evaluation Clinic based on predefined inclusion/exclusion criteria; (2) Eligible candidates will receive comprehensive study information, with written consent obtained by one Good Clinical Practice (GCP)-certified investigator; (3) The trial will adhere to Declaration of Helsinki principles, SPIRIT checklist (see Supplementary File 1) and Institutional Review Board (IRB). And, consent education, dedicated coordinator, withdrawal data collection will be implemented for promoting participant retention and complete follow-up. DSMB monitoring, IRB-approved, voluntary participation with no care penalties.

### Who will take informed consent? {26a}

Eligible patients will receive a verbal explanation and notice of signing the informed consent form before gastroscopy by GCP-certified investigator. Signed consent forms will be collected before randomization and stored in a locked cabinet.

### Additional consent provisions for collection and use of participant data and biological specimens {26b}

No additional consent provisions for collection and use of participant data and biological specimens in ancillary studies.

## Interventions

### Explanation for the choice of comparators {6b}

This study uses 0.9% saline placebo as the comparator for the following reasons: (1) Avoids confounding from respiratory interactions with other sedatives (opioids or benzodiazepines), which are commonly used in clinical practice but may independently affect hypoxemia. (2) Reflects real-world scenarios where propofol monotherapy is standard care for sedation in gastroscopy. Directly tests whether low-dose esketamine provides added benefit over current practice. (3) Placebo-controlled design is ethically justifiable because all patients receive propofol (standard care) as the base sedative and the trial population (STOP-Bang ≥ 3) is at high risk for hypoxemia, making detection of incremental benefits clinically meaningful.

### Intervention description {11a}

The anesthesia assessment should be completed a few days before the gastroscopy at Anesthesiology Clinic according to ASA guidelines. Once admitted to the gastroscopy room, intravenous (IV) access will be established, standardized monitoring will be continuously performed, including electrocardiogram (ECG), noninvasive blood pressure (NBP), SpO_2_, respiratory rate (RR), and waveform capnography. Then, placed in a left lateral position, investigators will deliver preoxygenation via facemask at 6 L/min.

Sedation protocol: An initial bolus of propofol (1.0–2.0 mg/kg for patients > 70 years; 1.5–2.5 mg/kg for patients < 70 years) injected. Then, esketamine 0.25 mg/kg is injected for the patients in Group E, while equal-volume saline solution is administered for Group C. Additional propofol (10–50 mg) boluses could be titrated until the palpebral reflex disappears or Modified Observer’s Assessment of Alertness/Sedation (MOAA/S) score of 2. During the procedure, additional propofol (10–50 mg) could be administered again for maintaining a MOAA/S score of 2 or addressing cough (≥ 2 consecutive coughs), grimaces (visible facial muscle contraction), involuntary body movements (limb/trunk movements requiring restraint), and hemodynamic instability (mean arterial pressure [MAP] increase ≥ 20 mmHg or heart rate [HR] increase ≥ 20 beat/min).

**Fig. 2:**
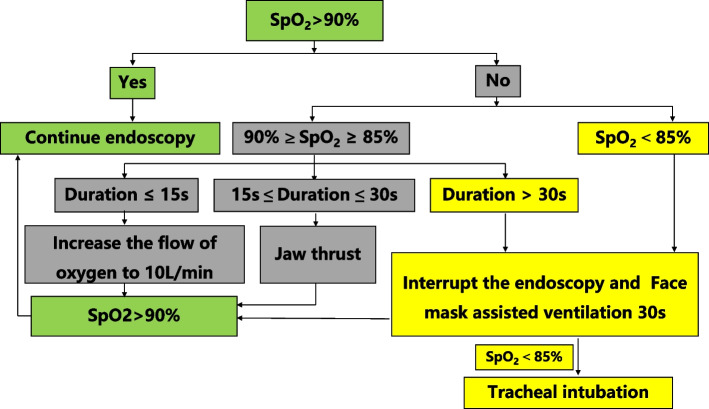
Airway management protocol

Emergency Airway Management: If the SpO_2_ dropped below 90% during the procedure, the airway management protocol in Fig. 2 will be implemented, redesigned according to the clinical practice and another completed trial [17]. If certain emergency situations occur, such as laryngeal spasm, an additional propofol (20–50 mg), methylprednisolone (40–80 mg, increasing the oxygen flow, gastroscope withdrawal, and positive pressure ventilation via the face mask, even rocuronium bromide 0.6 mg/kg and tracheal intubation if needed. No modification of randomized intervention (esketamine/saline); propofol dosing could be adjusted per clinical need (MOAA/S score).

Please consider revising the sentence " And in the emergency case of laryngospasm, an additional propofol (20–50 mg)" for clarity and completeness. Thank you, I change it.

Other adverse events (AEs) Management: In the case of HR < 50 beats/min, an intravenous injection of atropine (0.3–0.5 mg) will be administered. If the HR drops when NBP also decreases (a MAP 20% lower than at baseline), an intravenous injection of ephedrine (6–10 mg) would be administered and repeated as needed. If the HR is normal or above the normal range but with low NBP (< 90/60 mmHg or a MAP 20% lower than at baseline), deoxyadrenaline (50 ug) would be administered and repeated as needed.

### Criteria for discontinuing or modifying allocated interventions {11b}

Intervention may be stopped or modified if a participant experiences severe hypoxemia or other intolerable side effects. Worsening of the underlying condition requires alternative treatment. Incorrect randomization, eligibility errors, interim analysis result-related problems, and new safety data from other studies prompting modification or cessation need to discontinue or modify.

All substantial protocol modifications (changes to eligibility criteria, primary outcomes, or safety procedures) will be submitted for IRB review and approval prior to implementation, version-controlled in the protocol document (with revision dates), and updated on ChiCTR within 7 working days of approval.

### Strategies to improve adherence to interventions {11c}

To ensure strict adherence to the intervention protocol, we will implement a comprehensive strategy consisting of three key components: (1) standardized training for all study personnel using visual aids to reinforce proper procedures; (2) rigorous monthly audits by the Data Monitoring Committee (DMC) to review drug administration logs, titration compliance, and emergency protocol adherence; (3) a recognition system providing incentives for researchers who successfully complete 10 consecutive cases in full compliance with the study protocol. These measures are designed to maintain consistency in intervention delivery throughout the trial duration.

### Relevant concomitant care permitted or prohibited during the trial {11d}

Standard bowel prep, IV access, and rescue medications for hypotension/nausea are permitted, while sleep aids (7 days pre-op), additional sedatives/analgesics (opioids, benzodiazepines) during procedure/PACU stay, and elective surgeries within 72 h post-procedure are prohibited. All concomitant treatments will be documented in CRFs.

### Provisions for post-trial care {30}

Once upon gastroscope removal, sedation will be stopped. Patients with spontaneous respiration ability will be transferred to the PACU. Symptomatic supportive treatment according to clinical practice will be given, recording on CRFs and reporting to IRB.

### Outcomes and definitions {12}

Primary outcome is the hypoxemia incidence, defined as SpO_2_ < 90% for > 10 consecutive seconds during the procedure [18]. Timing begins at first SpO_2_ reading < 90% and continues until resolution.

Secondary outcomes are as follows:

1. Severe hypoxemia incidence, defined as SpO₂ ≤ 75% for any duration or SpO₂ ≤ 90% for ≥ 60 consecutive seconds or longer during the procedure [17].
2. Hypoxemia duration, defined as total cumulative time (seconds, mean [SD]/median [IQR]) with SpO₂ < 90% during procedure.
3. Emergency airway intervention, defined as requiring jaw thrust maneuver, mask-assisted ventilation with procedure interruption, tracheal intubation during procedure and in PACU.
4. Hypotension incidence defined as MAP decrease ≥ 20% compared with baseline (average of 3 pre-procedure readings) during procedure and in PACU.
5. Involuntary body movements are defined as any limb/trunk movements requiring physical restraint or procedure interruption during the procedure.
6. Total propofol dose defined as cumulative amount (mg, mean [SD]/median [IQR]) administered during procedure.
7. Length of procedure (minutes, mean [SD]/median [IQR]) defined as the time quantum from gastroscope insertion to complete withdrawal.
8. Recovery time (minutes, mean [SD]/median [IQR]) defined as the time quantum from scope removal to achieving MOAA/S score of 5 or responsive to normal voice.
9. Clinician satisfaction measured via 10-cm VAS immediately post-procedure (0 = extremely dissatisfied, 10 = completely satisfied) after the procedure.
10. AEs will be documented and assessed continuously during the procedure and in the PACU. Occurrence counts and severity (where applicable) will be documented in the CRFs as follows:
  Cough: Defined as ≥2 consecutive coughs during the procedure or in the PACU; assessed by direct observation and recorded as presence/absence.
  Hiccups: Defined as ≥2 episodes within 1 minute; recorded as presence/absence during the procedure or in the PACU.
  Nausea/vomiting: Graded on a 3-point severity scale (1=mild/nausea only, 2=moderate/transient vomiting, 3=severe/prolonged vomiting) and assessed both during the procedure and in the PACU.
  Reflux/aspiration: Defined as visible gastric content in the oropharynx or airway; recorded as presence/absence during the procedure.
  Laryngospasm: Defined as complete airway obstruction with no airflow despite respiratory effort; assessed by auscultation and waveform capnography, and recorded as presence/absence during the procedure.

### Participant timeline {13}

**Fig. 3:**
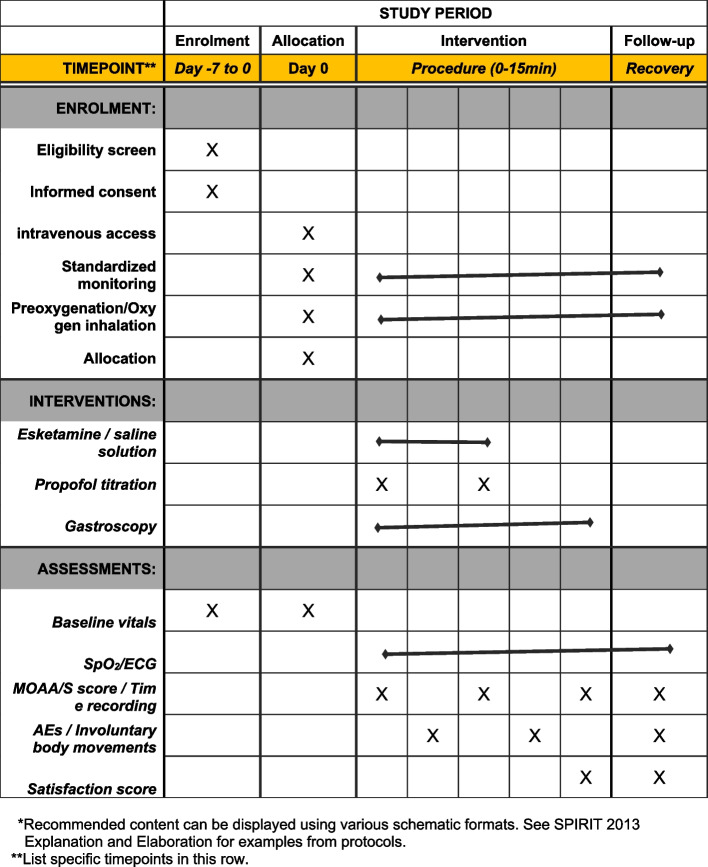
Time schedule of enrolment, interventions, and assessments

A detailed timeline of enrolment, interventions (including any run-ins and washouts), assessments, and visits for participants is shown in Fig. 3.

### Sample size {14}

All calculations were performed using PASS 15.0 (NCSS, LLC), with parameters verified by an independent statistician. The sample size calculation was based on detecting a clinically meaningful difference in the primary outcome of hypoxemia incidence (SpO_2_ < 90% for > 10 s) between study groups. From our pilot data, we anticipate a 50% incidence of hypoxemia in the control group receiving standard propofol sedation. We hypothesize that low-dose esketamine will achieve an absolute risk reduction of 15%, corresponding to a 35% hypoxemia incidence in the intervention group. Using a one-tailed *Z*-test for proportions (unpooled) with 80% power (*β* = 0.20) and a significance level of *α* = 0.025 (selected because esketamine’s established respiratory safety profile makes increased hypoxemia biologically implausible; detecting non-inferiority/superiority is the sole clinical priority.), we calculated a minimum requirement of 132 participants per group. To account for potential 10% attrition, we will enroll 147 participants per group (*N* = 294 total). This conservative approach ensures adequate power even if the actual control group incidence varies between 45 and 55% or if dropout rates reach 15%. The one-tailed design is appropriate given esketamine's established respiratory safety profile and the clinical irrelevance of detecting increased hypoxemia risk with this intervention.

### Recruitment {15}

In order to achieve adequate participant enrollment to reach target sample size, we have strengthened the recruitment plan to ensure SPIRIT compliance by (1) implementing a two-step screening process (electronic schedule review + pre-anesthesia STOP-Bang assessment) supervised by a dedicated coordinator; and (2) establishing tiered contingency plans (weekend slots) triggered if enrollment falls below 80% of targets. Monthly DSMB reviews will monitor progress, with all procedures emphasizing voluntary participation.

## Assignment of interventions: allocation

### Sequence generation {16a}

The allocation sequence will be computer-generated by an independent biostatistician using stratified block randomization (SAS 9.4). Stratification will be based on two clinically relevant factors: age group (18–65 years vs. > 65 years) and OSA severity (STOP-Bang score 5–6 vs. ≥ 7). Variable block sizes (4, 6, 8) will be used to enhance unpredictability, with details stored in a password-protected file accessible only to the trial statistician and DSMB chair. This ensures allocation concealment and prevents selection bias.

Roles are strictly separated to maintain blinding and prevent bias: sequence generated by an independent biostatistician, enrollment and consent completed by GCP-certified investigator, and intervention assigned by unblinded anesthesia nurse for prepares and dispenses study drugs per SNOSE system. The structured approach aligns with CONSORT and SPIRIT guidelines, ensuring robust randomization and minimizing bias.

### Concealment mechanism {16b}

The complete randomization scheme (including block sizes) will remain concealed in a restricted-access file. Emergency unblinding materials (sealed envelopes) will be attached to each participant’s CRF, accessible only to the chief anesthesiologist in case of serious adverse events.

### Implementation {16c}

The randomization sequence will be implemented using sequentially numbered, opaque, sealed envelopes (SNOSE) containing group assignments (esketamine 0.25 mg/kg or saline placebo). These envelopes will be securely stored in a locked cabinet, accessible only to an unblinded anesthesia nurse (not involved in outcome assessment).

## Assignment of interventions: blinding

### Who will be blinded {17a}

This study will be conducted in a double-blind manner where the patients, endoscopists, anesthesiologists responsible for intraoperative care, and outcome assessors will all be blinded to the group assignment.

All study drugs will be prepared by an independent anesthesiologist who is not involved in the subsequent anesthesia management, data collection, or outcome assessment. This anesthesiologist will have access to the randomization schedule.

Both the esketamine and the placebo (0.9% normal saline) will be drawn up to identical volumes of 10 mL in identical 20-mL sterile syringes. The syringes will be wrapped in opaque foil to prevent any visual distinction between the two solutions. The wrapped syringes will be labeled only with the patient’s study identification number.

The prepared, blinded syringe will be handed to the procedural anesthesiologist immediately before the initiation of anesthesia. This anesthesiologist will administer the study drug as a bolus without knowing its content.

### Procedure for unblinding if needed {17b}

Emergency unblinding is permitted only for suspected serious adverse reactions (SAEs). The principal investigator (PI) will hold the randomization list and be responsible for unblinding if necessary. All such events will be documented within 24 h to the IRB. Sealed envelopes attached to CRFs will reveal allocation.

## Data collection and management

### Plans for assessment and collection of outcomes {18a}

All outcomes will be systematically assessed, collected, and recorded on CRFs by blinded assessors across three phases: baseline demographics (age, sex, BMI), clinical characteristics (ASA status, STOP-Bang score, Difficulty in mask ventilation [DMV] risk factors), and room-air SpO_2_ will be collected pre-procedure; continuous vital signs (HR/SpO₂/NBP recorded every 30 s), propofol requirements, hypoxemia episodes (SpO_2_ < 90% > 10 s), procedure/recovery times, and adverse events (graded by blinded assessors) collected intraprocedure; Satisfaction (10-cm VAS), PACU recovery metrics (MOAA/S = 5 time), and AEs (graded 1–3 for severity/causality) collected post-procedure.

### Plans to promote participant retention and complete follow-up {18b}

To ensure high retention rates and complete follow-up data collection, the following strategies will be implemented: (1) clear consent discussions with visual aids emphasize study importance; (2) fixed anesthesia team and real-time CRF completion minimize errors; (3) immediate PACU assessments precede discharge, with small compensation (meal vouchers) offered. Withdrawals are documented without care penalties, and all collected data are retained for ITT analysis. Quarterly DSMB reviews monitor retention rates, with protocol deviations logged for sensitivity analyses. All data will be recorded in CRFs. Missing data (< 5%) will use mean imputation, analyzed via both ITT and per-protocol approaches. CRFs are publicly accessible via trial registry (ChiCTR2500099420).

### Data management {19}

Data will be collected electronically using a password-protected system with built-in range checks and mandatory fields. Data will be entered into Research Electronic Data Capture (REDCap) with range checks by blinded staff, with 10% double-entry verification. Standardized coding will ensure consistency. The system features role-based access, two-factor authentication, and encryption. All data will be stored for 10 years post-study on secure, backed-up servers.

### Confidentiality {27}

All participant data will be handled with strict confidentiality measures, collected by designated anesthesiologists and follow-up physicians, immediately recorded in password-protected electronic CRFs. Original records (medical charts, consent forms, CRFs) stored securely for 10 years. Electronic data are encrypted and accessible only to study statisticians. De-identified data could be shared with DSMB for monitoring, with no public release until trial completion and publication. Physical documents are shredded after 10 years per hospital policy, and electronic archives are permanently deleted.

### Plans for collection, laboratory evaluation and storage of biological specimens for genetic or molecular analysis in this trial/future use {33}

Not applicable. No laboratory evaluation and storage of biological specimens for genetic or molecular analysis in this trial.

## Statistical methods

### Statistical methods for primary and secondary outcomes {20a}

All analyses will be conducted using SPSS 23.0 (IBM Corp) by a statistician blinded to group allocation. The primary outcome will be analyzed using a one-sided *Z*-test at *α* = 0.025, while secondary outcomes will employ two-sided tests with *α* = 0.05.

Continuous variables will be assessed using the Kolmogorov–Smirnov test examining for normality. Normally distributed data will be presented as mean ± standard deviation (SD). Non-normal data will be presented as median or interquartile range (IQR). Categorical variables will be presented as counts and percentages.

For the primary outcome of hypoxemia incidence, we will employ a one-sided Z-test for proportions to evaluate the hypothesized reduction in the esketamine group, reporting absolute risk differences with 95% confidence intervals. Secondary outcomes will be analyzed using appropriate statistical tests based on variable characteristics: continuous normally distributed variables will be compared using independent *t*-tests, while non-normal continuous data will be assessed with Mann–Whitney *U* tests. Categorical outcomes will be analyzed using *χ*^2^ tests or Fisher’s exact tests as warranted by sample sizes. For time-to-event outcomes such as duration of hypoxemic episodes, we will utilize Kaplan–Meier survival analysis with log-rank tests. Longitudinal physiological measurements including mean arterial pressure and heart rate will be evaluated using repeated measures ANOVA, with post hoc Bonferroni corrections applied to account for multiple comparisons while maintaining the overall type I error rate at *α* = 0.05. All comparative analyses will be conducted by a blinded statistician using SPSS version 23.0, with two-sided tests (*α* = 0.05) employed for all secondary outcomes to conservatively assess potential group differences.

We will first conduct univariate screening of all clinically relevant variables, retaining those showing potential associations (*p* ≤ 0.10) for further analysis. Multicollinearity among candidate variables will be rigorously assessed using variance inflation factors (VIF > 2.5 indicating concerning collinearity) and examination of pairwise correlation coefficients (> 0.7 suggesting strong linear relationships). For multivariable analysis, we will employ backward stepwise logistic regression to identify independent predictors, with model selection guided by three key criteria: (1) clinical relevance of variables based on existing literature and physiological plausibility, (2) statistical significance of associations, and (3) overall model fit as evaluated by the Hosmer–Lemeshow goodness-of-fit test. Final models will report adjusted odds ratios with corresponding 95% confidence intervals to quantify the strength and precision of identified associations. This systematic approach ensures our regression analyses balance statistical rigor with clinical interpretability while appropriately addressing potential confounding relationships. No additional analyses.

All randomized participants will be analyzed according to ITT principles, maintaining original group allocations to preserve randomization benefits. A secondary per-protocol analysis will include only participants who completed ≥ 80% of study interventions without major protocol deviations, ensuring evaluation of treatment efficacy under ideal conditions. This dual approach balances real-world applicability with protocol fidelity assessment.

### Interim analyses {21b}

When half of the cases are completed in each group, unblinding and interim analysis will be conducted by an independent statistician. In the middle of the trial, that is, when half of the participants are enrolled, the test will be continued after unblinding under the condition that the interim analysis is consistent with the hypothesis. If it is inconsistent or even contrary, whether to continue the trial, terminate the trial, or expand the sample size will be decided by the expert committee.

### Methods for additional analyses (e.g., subgroup analyses) {20b}

Not applicable. No additional analyses in this protocol.

### Methods in analysis to handle protocol non-adherence and any statistical methods to handle missing data {20c}

The primary analysis will follow the ITT principle, including all randomized participants regardless of adherence to maintain randomization benefits. Sensitivity analyses (per-protocol, as-treated, or instrumental variable methods) will assess robustness for major protocol deviations or treatment switches. Missing data will be addressed using multiple imputation or mixed-effects models under the missing-at-random assumption. Sensitivity analyses (worst-case imputation) will evaluate the impact of missing data, with explicit reporting of assumptions and comparisons between complete-case and imputed results.

### Plans to give access to the full protocol, participant level-data and statistical code {31c}

The final trial protocol (including amendments) will be made publicly available upon study completion via Chinese Clinical Trial Registry, open-access journal, or email connecting with the corresponding author. Deidentified individual participant data, the protocol, and analytic code will be shared per repository name policies. Requests require a signed Data use agreement and approved research proposal.

## Oversight and monitoring

### Composition of the coordinating center and trial steering committee {5d}

The trial will be overseen by three independent committees: (1) A Trial Steering Committee (meeting quarterly) comprising independent clinicians and statisticians to provide overall supervision; (2) An independent Endpoint Adjudication Committee to verify primary outcomes; (3) Day-to-day operations will be managed by the Coordinating Center at Beijing Friendship Hospital, responsible for participant enrollment, data collection, and protocol implementation. Rigorous safety monitoring includes: mandatory reporting of all adverse events (graded 1–3 for severity/causality) to the IRB within 48 h; quarterly independent audits of protocol/GCP compliance; and immediate notification of all stakeholders for any protocol amendments. Results will be disseminated through peer-reviewed publications and clinical trial registries, with all committees maintaining independence from the sponsor. This multi-layered oversight framework ensures both trial integrity and participant safety throughout the study duration.

### Composition of the data monitoring committee, its role and reporting structure {21a}

The independent data monitoring committee (DMC) comprises statisticians, clinical experts, and ethics representatives, unaffiliated with the sponsor and investigator team, will monitor progress and protocol adherence, review data safety and interim analyses, and report directly to the sponsor’s governance board or independent ethics committee (not the study team). Interim findings are masked to investigators unless critical safety concerns arise.

### Adverse event reporting and harms {22}

All predefined sedation-related AEs (hypoxemia, hypotension) will be recorded in CRFs with severity grading (1–3), assessed for causality (Definite/Probable/Possible), and reported to IRB within 48 h (24 h for SAEs). Any participant experiencing trial-related adverse events/harms will receive immediate medical management, free follow-up care until resolution of symptoms, referral to appropriate specialists if needed, and some compensation according to hospital Compensation Policy.

### Frequency and plans for auditing trial conduct {23}

The trial will undergo independent audits every 3 months to ensure protocol compliance and data quality. Conducted by GCP-certified auditors unaffiliated with the study team or sponsor, these audits will include: (1) unannounced on-site inspections of study procedures; (2) random verification of 10% CRFs against source documents; and (3) assessment of AE reporting completeness. Audit reports will be submitted to both the DSMB and IRB within 14 days, with corrective action plans implemented for any findings. This process maintains investigator/sponsor independence while meeting SPIRIT and GCP standards for trial oversight.

### Plans for communicating important protocol amendments to relevant parties (e.g., trial participants, ethical committees) {25}

Protocol amendments (changes to eligibility, interventions, or risks) will be submitted to ethics committees and regulatory authorities for approval before implementation. All approved protocol amendments will be promptly updated on public registries within 30 days of regulatory/ethics approval. Participants will be re-consented if changes impact trial participation.

#### Dissemination plans {31a}

Trial outcomes will be disseminated through peer-reviewed publication in an open-access journal with public results tab. No publication restrictions apply and negative/null results will be reported. Lay-summary reports will be provided to all participants upon trial completion. After publication, the full protocol will be publicly accessible on ChiCTR. Detailed data and statistical documents could be available upon reasonable request via E-mail to the corresponding author.

## Discussion

This single-center, prospective, randomized controlled trial rigorously investigates the potential of low-dose esketamine (0.25 mg/kg) to mitigate propofol-induced hypoxemia in moderate-to-high risk OSA patients undergoing painless gastroscopy. Our study design addresses critical gaps in current sedation practices for this vulnerable population (patients with moderate-to-high risk OSA) by systematically evaluating whether low-dose esketamine reduces hypoxemia incidence while maintaining hemodynamic stability during procedural sedation.

The high incidence of hypoxemia (26–85%) during propofol sedation in OSA patients arises from multifactorial pathophysiology [19–21], including anatomical airway obstruction, sedative-induced respiratory depression, chronic pharyngitis, lung compression, and procedural factors. While airway optimization remains fundamental, our focus on pharmacological adjuvants represents a novel approach to this persistent clinical challenge. The selection of esketamine is particularly promising given its unique pharmacodynamic profile that may counteract propofol’s respiratory depressive effects while maintaining adequate sedation [14, 15].

Several methodological strengths enhance the validity of our trial design. First, our use of STOP-Bang scoring (≥ 3) provides a validated, clinically practical method for OSA risk stratification [22, 23], with clear correlation to polysomnography-confirmed disease severity. Second, we have implemented objective, clinically relevant endpoints aligned with international sedation guidelines [24], including both incidence and duration metrics for hypoxemia. Third, our protocol incorporates robust safety measures, including strict gastroscopy procedure time limits (≤ 15 min) based on our previous findings [25] and comprehensive monitoring of laryngospasm as a potential indicator of esketamine’s airway protective effects.

Notably, our secondary outcomes will provide important insights into esketamine’s multidimensional effects, including the following: propofol-sparing potential and hemodynamic stability [16], reduction in involuntary movements through analgesic properties, clinician satisfaction and procedural conditions, and recovery characteristics. These assessments may help explain the mechanistic basis for any observed hypoxemia reduction while evaluating the overall clinical utility of this adjunctive approach. Our preliminary data in urologic procedures [16] and analogous studies in other contexts [26] support the biological plausibility of our hypothesis.

Potential limitations include the single-center design and focus on gastroscopy, which may affect generalizability. However, the standardized protocol and objective endpoints strengthen internal validity. Furthermore, our stratified randomization and rigorous monitoring protocols minimize confounding and ensure data quality.

This trial will provide high-quality evidence regarding esketamine’s role in improving sedation safety for OSA patients. Positive results could establish a new standard of care for this high-risk population, while negative findings would still advance our understanding of sedation pharmacology in compromised airways. Either outcome will meaningfully inform clinical practice and future research directions in procedural sedation.

## Trial status

The first participant was enrolled on April 1, 2025; the recruitment will be completed on December 31, 2026. The trial is ongoing.

## Data Availability

After the study is published, the final trial dataset will be open to the public (including the participates) through email connecting with the corresponding author.

## Abbreviations

AEs/ASA: Adverse Events/ American Society of Anesthesiologists
BMI/CRF: Body / Case Report Form
ChiCTR: Chinese Clinical Trial Registry
DBP/MAP: Diastolic Blood Pressure/ Mean Arterial Pressure
DMC/DSMB: Data Monitoring Committee/ Data and Safety Monitoring Board
ECG/SpO₂: Electrocardiogram/ Peripheral Oxygen Saturation
GCP: Good Clinical Practice
HR/RR: Heart Rate/ Respiratory Rate
IRB: Institutional Review Board
ICMJE: International Committee of Medical Journal Editors
ITT/IV: Intention-to-Treat/ Intravenous
MOAA/S: Modified Observer’s Assessment of Alertness/Sedation
NBP/SBP: Noninvasive Blood Pressure/ Systolic Blood Pressure
NMDA: N-Methyl-D-Aspartate (receptor)
OSA: Obstructive Sleep Apnea
PACU: Post-Anesthesia Care Unit
SNOSE: Sequentially Numbered, Opaque, Sealed Envelopes
STOP-Bang: Snoring, Tiredness, Observed apnea, Pressure (blood), BMI, Age, Neck size, Gender
VAS: Visual Analog Scale
